# Motor Learning Improvement Remains 3 Months After a Multisession Anodal tDCS Intervention in an Aging Population

**DOI:** 10.3389/fnagi.2018.00335

**Published:** 2018-10-24

**Authors:** Gaëlle Dumel, Marie-Eve Bourassa, Camille Charlebois-Plante, Martine Desjardins, Julien Doyon, Dave Saint-Amour, Louis De Beaumont

**Affiliations:** ^1^Centre de Recherche de l’Hôpital du Sacré-Coeur de Montréal, Montréal, QC, Canada; ^2^Département de Psychologie, Université du Québec à Montréal, Montréal, QC, Canada; ^3^Unité de Neuroimagerie Fonctionnelle, Centre de Recherche de l’Institut de Gériatrie de Montréal, Montréal, QC, Canada; ^4^Département de Chirurgie, Université de Montréal, Montréal, QC, Canada

**Keywords:** brain plasticity, aging, non-invasive brain stimulation, motor cortex, motor learning, durability, rehabilitation

## Abstract

Healthy aging is associated with decline of motor function that can generate serious consequences on the quality of life and safety. Our studies aim to explore the 3-month effects of a 5-day multisession anodal transcranial direct current stimulation (a-tDCS) protocol applied over the primary motor cortex (M1) during motor sequence learning in elderly. The present sham-controlled aging study investigated whether tDCS-induced motor improvements previously observed 1 day after the intervention persist beyond 3 months. A total of 37 cognitively-intact aging participants performed five consecutive daily 20-min sessions of the serial-reaction time task (SRTT) concomitant with either anodal (*n* = 18) or sham (*n* = 19) tDCS over M1. All participants performed the Purdue Pegboard Test and transcranial magnetic stimulation (TMS) measures of cortical excitability were collected before, 1 day after and 3 months after the intervention. The last follow-up session also included the execution of the trained SRTT. The main findings are the demonstration of durable effects of a 5-day anodal tDCS intervention at the trained skill, while the active intervention did not differ from the sham intervention at both the untrained task and on measures of M1-disinhibition. Thus, the current article revealed for the first time the durability of functional effects of a-tDCS combined with motor training after only 5 days of intervention in an aging population. This finding provides evidence that the latter treatment alternative is effective in achieving our primary motor rehabilitation goal, that is to allow durable motor training effects in an aging population.

## Introduction

In the last two decades, there has been a growing interest in using anodal transcranial direct current stimulation (a-tDCS) in order to increase corticomotor excitability and associated functions such as motor learning (Muellbacher et al., 2000; Sanes and Donoghue, 2000; Fregni and Pascual-Leone, 2007; Galea and Celnik, 2009; Reis et al., 2009; Fritsch et al., 2010; Stagg et al., 2011; Ditye et al., 2012). Interestingly, despite the significant age-related decline of primary motor cortex (M1) excitability and function (Nitsche et al., 2008; Fathi et al., 2010), numerous studies reported motor function gains associated with the application of a-tDCS over M1 in an aging population (a-tDCS-M1; Zimerman et al., 2013; Parikh and Cole, 2014; Hoff et al., 2015; Panouillères et al., 2015; Dumel et al., 2016). This finding highlights the potential relevance of a-tDCS-M1 in the latter population considering that declining motor function in aging is associated with serious consequences on the quality of life and safety of the elderly. Nevertheless, there is still limited information about the long-term aftereffects and durability of a-tDCS-M1 intervention in aging.

First of all, a critical element in evaluating the clinical utility of any therapeutic intervention is the durability of the observed effects. Single-session tDCS interventions aftereffects are generally short lived and functional benefits have not been steadily reproducible, which considerably hinders its therapeutic value (Horvath et al., 2015). However, multisession tDCS protocols have proven to induce more reliable and durable gains in young healthy subjects (Fregni et al., 2006; Alonzo et al., 2012; Ditye et al., 2012; Gálvez et al., 2013; Meinzer et al., 2013). For instance, a significant cumulative increase in cortical excitability was found in young healthy individuals with the application of a-tDCS-M1 when applied daily for 20 min over five consecutive days (Gálvez et al., 2013). A similar study found that continuous a-tDCS at 2 mA for 20 min induced changes in M1 excitability that lasted for at least 2 h, with a linear increase in excitability when sessions were repeated on a daily basis over a 5-day period (Alonzo et al., 2012).

Moreover, it is generally agreed that a-tDCS-dependent behavioral gains are optimized with concurrent behavioral training (Fregni and Pascual-Leone, 2007; Galea and Celnik, 2009; Reis et al., 2009; Stagg et al., 2011; Ditye et al., 2012). For example, the application of a-tDCS-M1 during the execution of an explicit sequence-learning task was associated with faster learning, compared with either online sham stimulation or offline a-tDCS-M1 stimulation (Stagg et al., 2011).

Taken together, these results suggest that the application of multisession tDCS protocols during motor training can accentuate and maintain behavioral gains in healthy adults. Accordingly, another study revealed that young adults who received three consecutive, daily 20-min sessions of a-tDCS-M1 during a sequential finger tapping task showed significantly greater motor learning relative to a sham control group (Saucedo-Marquez et al., 2013). Similarly, in young healthy controls, five daily consecutive 20-min sessions of a-tDCS-M1 combined with a motor learning task were associated with online task performance improvements that persisted over 3 months after the intervention (Reis et al., 2009).

In aging, to our knowledge, only two multisession tDCS studies combined with cognitive training were conducted and they both showed long-term aftereffects (Park et al., 2014; Jones et al., 2015). However, these studies targeted the dorsolateral prefrontal cortex and not M1 so that the long-term aftereffects of multisession a-tDCS protocols involving M1 in an aging population is unknown.

Another crucial element in determining the utility of any rehabilitation intervention is the generalizability of training gains to untrained tasks. Although the generalization of motor skills after motor training remains poorly understood, generalizability of training gains appears to be achievable in conditions where extensive training over multiple sessions is undertaken (King et al., 2013; Boraxbekk et al., 2016). Accordingly, learning transfer of a trained to an untrained finger tapping sequence was observed after 6 weeks of training in a recent aging study (Boraxbekk et al., 2016). In the same vein, others aging studies showed improvements in manual dexterity, as measured with the Purdue Pegboard Task (PPT), after motor training involving three sessions per week over 6 weeks (Kornatz et al., 2005) and five sessions per week over 8 weeks (Ranganathan et al., 2001). Interestingly, a recent sham-controlled aging study from our group showed generalization effects on the untrained PPT only in the group who received a-tDCS-M1 stimulation during motor training for a total of five consecutive, daily 20-min sessions (Dumel et al., 2018). This finding suggests that the online application of a-tDCS-M1 during motor training considerably accelerated the generalization of motor learning to an untrained task.

In addition to facilitating motor learning generalization, multisession a-tDCS-M1 in aging was found to allow disinhibition of long-interval cortical inhibition (LICI) of M1 (Dumel et al., 2018) as measured with transcranial magnetic stimulation (TMS), a technique often used to characterize plasticity-dependent cortical excitability changes induced by a-tDCS-M1 stimulation (Wassermann et al., 2008). Interestingly, the latter LICI disinhibition of M1 correlated with motor performance during intervention as well as with motor learning generalization. These results are in line with previous studies that showed significant motor and cognitive improvements in young adults with the activation of GABA-b receptors (Mondadori et al., 1996; Flood et al., 1998; Getova and Bowery, 1998; Escher and Mittleman, 2004; Froestl et al., 2004; Helm et al., 2005), the latter being associated with LICI modulation (McDonnell et al., 2006). While the aging process is known to modify GABAergic neurotransmission (Levin et al., 2014) in a way that is associated with motor dysfunctions (Gleichmann et al., 2011), these findings highlight the therapeutic potential of targeting GABA-b receptors activity to improve aged-related motor deficits.

Here, we investigated whether a five-session a-tDCS-M1 intervention conducted in a healthy aging population would be associated with durable motor generalization effects when tested 3 months later. To this end, we contrasted baseline M1-disinhibition and motor performance on a trained (i.e., Serial Reaction Time Task, SRTT) and an untrained (i.e., PPT) task with data collected at 1 day and 3 months after the completion of the a-tDCS-M1 intervention in a sham-controlled study design. We hypothesized that relative to cognitively-intact aging individuals assigned to the sham control group, age-equivalent individuals from the a-tDCS group would still exhibit greater performance on both trained and untrained tasks at 3 months post-intervention. We also sought to test whether M1 disinhibition found within 24 h of the conclusion of the intervention would persist when assessed 3 months later.

## Materials and Methods

### Participants

All 37 participants (61 ± 6, 28 years-old; range, 51–74 years, 19 women) were healthy, right-handed elderly adults recruited via newspaper ads. All of them were submitted to the same intervention. A previous study from our laboratory (Dumel et al., 2016), which focused strictly on a-tDCS effects on SRTT training, presented data collected with a total of 23 of the 37 subjects. Continued recruitment allowed us to publish a second study from our laboratory that presented results obtained from 32 of the current 37 subjects to investigate motor generalization effects within 24 h of completion of the 5-day a-tDCS-M1 intervention (Dumel et al., 2018). In the current study, which included five new participants, we investigated the durability of a-tDCS effects on motor function and cortical excitability using a SRTT, a PPT and standard TMS measures taken at 3 months post intervention. Data collected at 3 months have not been used in our previous study or elsewhere.

Participants were first submitted to a phone interview and were included if they met all of the following self-reported criteria: good general health including no significant neurological history (e.g., traumatic brain injury, stroke, encephalopathy, seizure disorder); no history of alcohol and/or substance abuse; no psychiatric illness or learning disability. Inclusion and exclusion criteria were thoroughly verified at the beginning of the first visit. None of them reported using centrally acting drugs, having movement restriction or pain in their right arm or hand, or regularly practicing any activity that involved repeating sequential finger movements (e.g., playing a musical instrument or video games). Participants were also screened for cognitive impairment and depression using the Mini-Mental State Examination (MMSE; Folstein et al., 1975) and the Beck Depression Inventory II (BDI-II; Beck et al., 1996) with cut-offs of 27 and 13, respectively (see Table 1). This study was carried out in accordance with the recommendations of “Santé Canada” and the “Comité d’éthique de la recherche et de l’évaluation des technologies de la santé de l’hôpital Sacré-Coeur de Montréal” with written informed consent from all subjects. All subjects gave written informed consent in accordance with the Declaration of Helsinki. The protocol was approved by the “Comité d’éthique de la recherche et de l’évaluation des technologies de la santé de l’hôpital Sacré-Coeur de Montréal.”

**Table 1:** Groups.

	Anodal	Sham	*t*	*P*
*N*	18	19	−	−
Male/Female	9/9	9/10	−	−
Age	61.56 ± 5.85	61.26 ± 6.82	0.140	0.890
Education	16.61 ± 2.52	17.58 ± 2.73	−1.117	0.272
BDI score	2.50 ± 2.64	3.17 ± 3.11	−0.693	0.493
MMSE score	29.22 ± 1.06	29.11 ± 1.05	0.337	0.738

*Mean ± Standard Deviation*.

This study was approved by the Research Ethics Committee of the *Hôpital du Sacré-Coeur de Montréal*, and all participants provided written informed consent before testing. Participants received a financial compensation for their participation.

Participants were assigned to one of two groups via a stratified randomization procedure; an anodal tDCS group (*n* = 18) and a sham-stimulation group (*n* = 19). The two groups were closely matched in terms of gender distribution, age and level of education (see Table 1).

Given the known effects of sleep on learning, the subjects’ sleep quality on the night preceding testing was assessed at the beginning of each session. Participants were asked to evaluate the quality of their sleep (on a scale ranging from very bad to very good), their mood when waking up (on a scale ranging from very tense to very calm) and their level of vigilance when waking up (on a scale ranging from very tired to very awake) by drawing an intersecting line on a 10-cm visual analog scale. The maximal score was fixed at 10, where each point corresponded to 1 cm on the scale. Averaged sleep quality on the night before the 5 tDCS interventions was equivalent across groups (*t*_(1,35)_ = 0.290; *p* = 0.774) as well as sleep quality of the night before the 3 months follow-up session (*t*_(1,33)_ = −1.552; *p* = 0.130).

### Study Design

The experiment involved eight sessions each conducted on separate days, which included a motor training intervention over five consecutive days (D1 to D5) as well as pre-post outcome measures collected 1 day before training (Pre), 1 day after training (Post) and 3 months later (Post3). The intervention sessions consisted of 20-min training sessions involving the execution of a modified SRTT, where half of subjects received concomitant anodal tDCS stimulation, while the other half was exposed to a sham stimulation. Sessions took place between 8 a.m. and 5 p.m. and were separated by 24 h. The time of day of testing was kept constant throughout the five sessions for each participant and was equivalent between both groups. Each intervention session lasted approximately 40 min. Pre-post intervention sessions lasted about 90 min each, including the assessment of manual dexterity with the PPT, followed by a TMS-based assessment of M1 excitability with various TMS protocols including LICI assessment. The last session, scheduled to take place 3 months after the intervention, included the execution of the trained SRTT in addition to the PPT and TMS protocols.

### Intervention

#### tDCS

A-tDCS was delivered through two saline-soaked sponge electrodes (7.5 cm × 6 cm) connected to a constant direct current stimulator (*HDCKit*, Newronika, Milan, Italy). We used a bipolar electrode montage with a 2 mA direct current flowing from an anode positioned over the left M1 to a reference electrode positioned on the contralateral supraorbital area (Nitsche and Paulus, 2000). For precise and individualized localization, the left M1 hand area was identified in all subjects at the vertex using TMS and was kept constant across intervention sessions using a 3D stereotaxic TMS manager device (*Northern Digital Instruments*, Waterloo, ON, Canada). In the anodal group, the stimulation was applied continuously for 20 min throughout each motor training session. By contrast, the same installation was used in the sham group, but the current was interrupted after having completed the initial 30-s ramp up and ramp down. Only the investigator was aware of the type of stimulation (anodal or sham).

#### Trained Skill

During tDCS application, participants performed a custom SRTT running on MatLab (version R2012b; The MathWorks, Natick, MA, USA) and designed to measure implicit motor sequence learning (Duchesne et al., 2015). Each trial consisted of one filled yellow circle and three white circles of equal size (3.6 cm diameter), positioned at an equal distance in an inverted U shape. The position of the target (yellow circle) varied across trials among the four possible locations and indicated the correct key press. Participants were instructed to respond as fast and accurately as possible to the position of the yellow circle by pressing the corresponding key on the game board (model G13; Logitec, Lausanne, Switzerland) with the appropriate predetermined fingers of the right hand throughout the entire task. Participants performed a total of 30 blocks separated by 15-s pauses, including 10 random (R) and 20 sequence (S) blocks of trials. The 10 R-blocks were inserted among the S-blocks as follows: S-blocks, R-blocs, S-blocks, S-blocks, R-blocs, S-blocks etc. Each block included 60 trials, i.e., 60 appearances of a yellow circle. Each yellow circle remained on the screen until a key press was made (correct or incorrect) and was immediately replaced by the next trial. Each of the 20 sequence blocks consisted of five presentations of the same 12-item sequence. In order to make sure that motor sequence learning remained implicit over five consecutive sessions, distinct but equivalent 12-item sequences were presented on each of the 5 tDCS sessions for each participant (refer to Dumel et al., 2016 for information about sequences) for information about sequences). Each session began with a random practice block (60 trials). Response time (RT) was defined as the time interval between stimulus presentation (yellow circle) and the key press response. Sequence-specific learning (percent change in RT) per day of training was computed as follows: ((*mean RT R-blocks − mean RT S-blocks*)/*mean RT R-blocks*) × 100. This measure allows to dissect sequence-specific learning while controlling for familiarity with the task procedure for any given day of training.

### Pre-post Intervention Outcome Measures

#### Trained Skill

To assess motor training persistence at 3-month post-intervention, we administered the third (day 3) 20-min SRTT training session sequence of the 5-day intervention without the tDCS montage.

#### Untrained Skill

The PPT (*Lafayette Instrument*, Model 32020, Lafayette, IN, USA; ICCr = 0.632) was used to measure bimanual and unimanual dexterity, i.e., an untrained M1 function. The pegboard has two parallel columns of 25 holes into which participants have to insert as many cylindrical metal pegs as possible in 30 s, starting from the top toward the bottom of the columns. The first condition is performed with the right hand, the right column of holes and the metal pegs container on the right-hand side of the board. The second condition is performed with the left hand using the left column and container. The third condition is performed with both hands simultaneously. Each condition is performed three times, for a total duration time per condition of 90 s. The number of metal pegs (conditions 1 and 2) or pairs of metal pegs (condition 3) inserted in 30 s was recorded. The averaged number of inserted metal pegs was calculated for each condition. Importantly, as the temperature can influence the performance of the task (Muller et al., 2011), the ambient temperature in the room was maintained at 22 ± 1°C.

#### M1-Disinhibition

To assess left M1 cortical disinhibition, we used TMS generated via a dual-pulse Magstim 200 magnetic stimulator (*Magstim Company*, Whitland, Dyfed, UK) and a figure-of-eight coil with 80 mm wing diameter. Participants were asked to sit on a comfortable chair while the experimenter positioned the coil over the scalp. Three electrodes were affixed on the right hand of participants to record the MEPs of the first dorsal interosseus (FDI) muscle throughout the testing session. Each session began with determining the exact location of the vertex over the left M1, i.e., the site inducing reliable, maximal peak-to-peak amplitude MEPs of the FDI. This site was recorded using a 3D tracking system (*Northern Digital Instruments*, Waterloo, ON, Canada) to ensure consistent coil positioning throughout the four distinct TMS protocols. Participants first underwent a single-pulse resting motor threshold (rMT) paradigm. RMT was determined as the minimal stimulation intensity evoking a MEP in the resting FDI of at least 50 mV in 6 out of 10 consecutive trials with an interpulse interval of 8–10 s. We then conducted various paradigms to further investigate M1 excitatory and inhibitory mechanisms. LICI was obtained via a dual-pulse paradigm where 2 TMS stimulations of identical intensity (i.e. 120% of the rMT) were separated by a 100-ms interstimulus interval. When LICI is conducted in healthy adults, the first pulse inhibits the amplitude of the second MEP induced by the second TMS pulse. The LICI variable is calculated as the ratio of the *second MEP peak-to-peak amplitude* relative to the* first MEP peak-to-peak amplitude*.

### Statistical Analyses

First of all, a Kolmogorov-Smirnov test revealed that the distribution of the data on the primary outcome measures (difference between reaction times at 3 months and first day of motor training) was normal (*p* = 0.16). To evaluate the durability of tDCS effects on trained SRTT skill, we average reaction times of sequence blocks completed on the first day of intervention with those completed on the Post3 session after having combined both groups. We then computed an independent samples *t*-test analysis to contrast group performance on sequence-specific learning at the Post3 session. The durability of tDCS intervention effects on untrained PPT skill (number of pegs inserted) and M1-dishinbition (LICI ratios) were evaluated in comparing group results across two-time windows: pre-intervention using a series of ANCOVA tests: Group × Time (Baseline vs. Post), Group × Time (Baseline vs. Post3) and Group × Time (Post3 vs. Post). As per our previous multisession a-tDCS study showing significant intervention effects on LICI ratios and not on other TMS protocols (Dumel et al., 2018), only LICI ratio changes at Post3 were investigated in this study.

## Results

### Trained Skill

**Figure 1:**
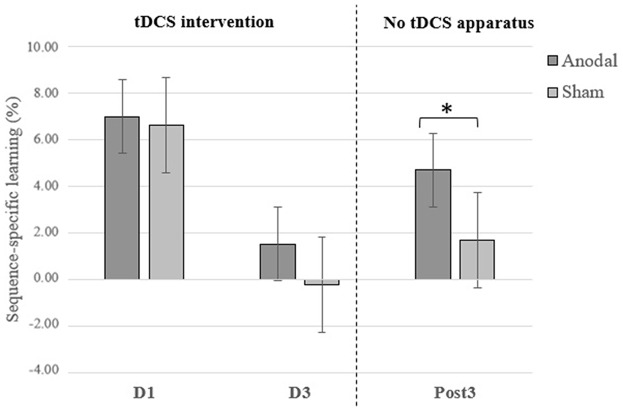
Sequence-specific learning at the first (D1) and the third (D3) days of tDCS intervention and 3 months later (Post3). Error bars = standard error. *p = 0.05.

**Figure 2:**
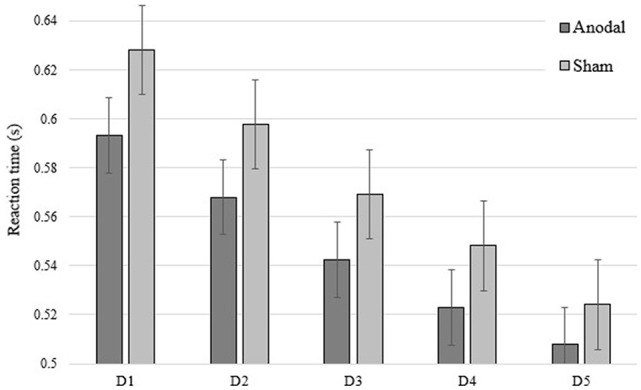
Overview of mean response time (RT; sequence and random blocks collapsed) per group and per day of intervention.

As expected, reaction time scores on sequence blocks at the Post3 session were significantly faster than on the first day of motor training when combining both groups (*F*_(35)_ = 75.55; *p* < 0.0001; $\eta_{\text{p}}^{2}$ = 0.737). This suggests a general SRTT skill maintenance over 3 months in both a-tDCS and sham groups. Importantly, the *t*-test revealed a significant between-groups difference on sequence-specific learning at the Post3 session (*t*_(34)_ = 2.030; *p* = 0.050; Cohen’s *d* = 0.69) and Levene test indicated that variances were homogeneous (*F* = 3.074; *p* = 0.089). This finding points to the durability of tDCS intervention effects on sequence-specific learning at the SRTT. Although not performed under the same testing conditions—namely the tDCS apparatus was not applied on the subject’s head in addition to not accounting for offline consolidation effects—it is interesting to note that the significant group difference obtained at Post3 session was neither found at Day 1 (*t*_(36)_ = 0.279; *p =* 0.782) nor at Day 3 (*t*_(36)_ = 1.54; *p =* 0.133) of the intervention (see Figure 1). This finding provides conjectural information that either 1 or 3 days of training with a-tDCS were insufficient to generate between-groups differences on sequence-specific learning at a SRTT. See Figure 2 for an overview of reaction times during the 5-day intervention.

#### Untrained Skill

**Figure 3:**
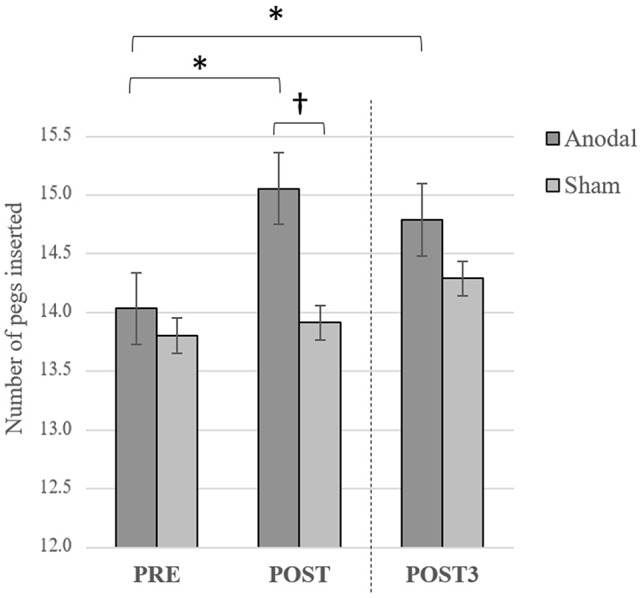
Averaged number of pegs inserted with the right hand computed over three consecutive trials on baseline (Pre), Post and Post3 for both groups. Error bars = standard error. *p < 0.05; ^†^*p* < 0.08.

**Figure 4:**
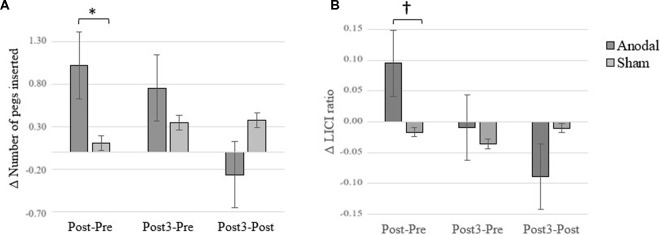
Changes between baseline (Pre) vs. Post and Post3 sessions. **(A)** Purdue Pegboard Task (PPT) performance. **(B)** M1-Desinhibition. Error bars = standard error. *p < 0.05; ^†^*p* < 0.10.

Consistent with a previous study from our group (Dumel et al., 2018), we found a significant Group × Time (Baseline vs. Post) interaction on the averaged number of pegs inserted with the right hand computed over three consecutive trials (*F*_(1,34)_ = 6.31; *p* = 0.017; $\eta_{\text{p}}^{2}$ = 0.153). However, a similar Group × Time interaction involving Baseline vs. Post3 for the same variable failed to reach statistical significance (*F*_(1,34)_ = 1.18; *p* = 0.284; $\eta_{\text{p}}^{2}$ = 0.034; see Figure 4). Group × Time interaction comparing Post3 vs. Post did not reveal to be significant (*F*_(1,34)_ = 0.146; *p* = 0.705; $\eta_{\text{p}}^{2}$ = 0.005). *Post hoc* independent samples *t*-test analysis on the averaged number of pegs inserted with the right hand before the intervention revealed to be comparable (*t*_(35)_ = 0.389; *p* = 0.700). While the independent samples *t*-test revealed a near significant group difference 1 day post intervention (*t*_(35)_ = 1.979; *p* = 0.056; Cohen’s *d* = 0.58), this trend was no longer observed at Post3 session (*t*_(34)_ = 0.870; *p* = 0.390). However, when we conducted one-sample *t*-test analyses to compare PPT performance for the anodal group only, we found a significant difference on baseline vs. Post (*t*_(17)_ = −3.505; *p* = 0.003; Cohen’s *d* = 0.60) as well as between baseline and Post3 (*t*_(17)_ = −2.34; *p* = 0.032; Cohen’s *d* = 0.44). None of the between-sessions PPT performance differences were found to be significant for the sham group (baseline vs. Post: *t*_(18)_ = −0.491, *p* = 0.629; baseline vs. Post3: *t*_(17)_ = −1.849, *p* = 0.082). These last results are illustrated in Figure 3.

#### M1-Disinhibition

While the ANCOVA for the Pre vs. Post LICI variable revealed a near significant Group × Time interaction (*F*_(1,34)_ = 3.17; *p* = 0.084; $\eta_{\text{p}}^{2}$ = 0.088), Pre vs. Post3 LICI changes did not differ across groups (*F*_(1,32)_ = 0.146; *p* = 0.705; $\eta_{\text{p}}^{2}$ = 0.005; see Figure 4). Group × Time interaction comparing Post3 vs. Post did not reveal to be significant (*F*_(1,34)_ = 1.936; *p* = 0.173; $\eta_{\text{p}}^{2}$ = 0.054). When we conducted *post hoc* one-sample *t-test* analyses on LICI at baseline vs. Post-intervention for the anodal group, we found a near significant difference (*t*_(15)_ = −2.03; *p* = 0.06), while the baseline and Post3 comparison did not reveal any difference (*t*_(14)_ = 0.345; *p* = 0.736). None of the between-sessions PPT performance differences were found to be significant for the sham group (baseline vs. Post: *t*_(18)_ = 0.402, *p* = 0.692; baseline vs. Post3: *t*_(17)_ = 0.599, *p* = 0.557). These results suggest that tDCS effects on LICI disinhibition did not persist at 3 months post-intervention.

## Discussion

The current sham-controlled study investigated whether durable multi-sessions a-tDCS-M1 effects on motor learning observed more than 3 months after training in young healthy controls (Reis et al., 2009) could be generated in a healthy aging population. The main findings are the demonstration of durable effects of a 5-day anodal tDCS intervention at the trained skill, while the active intervention did not differ from the sham intervention at both the untrained task and on measures of M1-disinhibition.

Present results show for the first time in an aging population that the facilitatory effects of a 5-day a-tDCS intervention on the trained motor skill persist beyond 3 months after the completion of the intervention. This finding is in line with a previous study conducted in young adults that showed maintenance of a 5-day tDCS effects on trained skill when measured at 3 months post-intervention in young adults (Reis et al., 2009).

Retention of motor skills over extended delays is well documented (Hovland, 1951; Fleishman and Parker, 1962; Arthur et al., 1998; Alonzo et al., 2012) and is in line with the long-standing observation that motor procedure such as riding a bicycle tend to persist over time, sometimes extending over decades. In keeping with this notion, results from this study also show significant performance improvements at the SRTT when tested 3 months later relative to the first day of training in both sham and a-tDCS groups, suggesting procedural long-term retention induced by task-specific training. Previous experiments noticed that reaction times improvements at an acquired motor skill are particularly time-resistant, as demonstrated in a series of studies showing effects lasting more than 1 year after training in a young cohort (Willingham and Dumas, 1997; Hikosaka et al., 2002). Importantly, the 3-month follow-up data from the present study shows that the application of a-tDCS-M1 allows a significantly greater long-time skill retention, as an added effect to the SRTT training alone derived from the sham control group. In other words, the added effect of motor learning generated through 5 days of intervention (Dumel et al., 2016, 2018) persist beyond a 3-month delay.

While a trend toward M1-desinhibition was measured on a LICI TMS protocol 1 day following intervention, the current study did not show any difference between the sham and the anodal stimulation groups at the latter measure when at Post3 session. This finding indicates that the known, practice-dependent effects of multi-session anodal tDCS combined with motor training on cortical excitability of M1 tend to diminish with time. In a recent article, Rahman et al. (2017) explored the cortical mechanisms underlying the coaction of local synaptic activation induced by tDCS and afferent activity induced by motor training. Their findings revealed that the likelihood of synaptic plasticity is increased by the coincidence of sustained tDCS effects on presynaptic firing and pre- and postsynaptic action potentials induced by training. Considering the practice-dependent effects of tDCS on the efficacy of cortical synapses, a reduction of this effect is to be expected after 3 months without practice nor stimulation.

Furthermore, the current study reveals an insignificant Groups × Time (baseline vs. Post3) interaction at the untrained PPT, a finding that is at odds with data collected within 24 h of intervention completion. Although not a specific aim of the current study, the latter finding suggests that motor skill transfer may depend on plasticity-dependent early stages of consolidation process facilitated by anodal tDCS (Tecchio et al., 2010; Reis et al., 2013). This interpretation is consistent with the significant correlation found at 1 day post-intervention between M1-desinhibition and motor generalization (Dumel et al., 2018). In parallel, the current study reveals a significant performance improvement at the PPT between baseline and Post3 measures specific to the anodal group. However, while not reaching significance levels, the same is observed in the sham group. Indeed, repetition of the task induced gradual performance improvements in individuals from the sham group across sessions, allowing them to almost join a-tDCS group level at the Post3 session.

The short- and long-term tDCS effects found herein can be understood as brain stimulation effects on two time-dependent consolidation types (Au et al., 2017). On one hand, fast-acting “synaptic consolidation” has been linked to changes of synaptic connections in localized neuronal circuits that occur within minutes to hours following a learning event (Born and Wilhelm, 2012; Au et al., 2017). It is well-established that a-tDCS plays a role in fast-acting consolidation processes considering its interactions with gene expression—namely BDNF (Podda et al., 2016)—and neurotransmitters receptors activation—namely GABA (Krause et al., 2013) and glutamate (Stagg and Nitsche, 2011)—generating enhancements of synaptic efficacy (Fritsch et al., 2010; Stagg and Nitsche, 2011; Rahman et al., 2013). Online and short-offline tDCS effects are thought to be the result of initial brain plasticity modulation implying long-term potentiation (Au et al., 2017). Second, remote effects on the trained task suggest that a-tDCS could act on “system consolidation.” Indeed, system consolidation refers to a slow-acting system that occurs in the order of days, months or even years following a learning event. System consolidation involves the “replay” of cells which were engaged at a synaptic level and the redistribution of learned information to the long-term store (Born and Wilhelm, 2012; Au et al., 2017). Although quite speculative at this stage, it has been hypothesized that in modulating cortical excitability (via glial-neuron interactions), a-tDCS enhances neural replay which would in turn strengthen consolidation effects (Au et al., 2017). Interestingly, this model would be consistent with the observation that a-tDCS-induced LICI disinhibition is observed only within 24 h of intervention completion.

The present study is not without limitations. First, the sample size is relatively small and participants recruited in this study are generally highly educated, which restrict the generalizability of the current study findings to a subset of the general aging population. In addition, the present study also raises numerous questions requiring further investigation. For instance, our protocol did not allow us to measure whether durable effects on the trained task persisted beyond 3 months of the intervention. In this respect, it is noteworthy to mention that reaction times improvements after extensive SRTT training, without tDCS, have been documented at 1 year after intervention in a young population (Willingham and Dumas, 1997; Hikosaka et al., 2002). Accordingly, Hikosaka et al. (2002) observed these effects after a daily SRTT training conducting over 8–10 days. In this regard, our current study design did not allow us to test whether additional tDCS sessions and training would have either extended the observed beneficial effects beyond 3 months on the trained task or would have been sufficient to generate significant durable effects at the untrained task. Finally, while it has never specifically been tested to our knowledge, several authors—studying treatment of depression or neuronal pain using tDCS intervention—raised the possibility that additional tDCS session conducted before the follow-up measurements might facilitate the maintenance of long-term aftereffects (Nitsche et al., 2009; Ngernyam et al., 2013). Along those lines, we suggest that future studies could implement such booster sessions to investigate the possibility of enhancing or maintaining a-tDCS/motor training effects in motor rehabilitation in aging.

In conclusion, the current article revealed for the first time the durability of functional effects of a-tDCS combined with motor training after only 5 days of intervention in an aging population. In addition to the significant short-term effects on trained task and motor generalization (Dumel et al., 2016, 2018), the current findings provide evidence that the latter treatment alternative is effective in achieving our primary motor rehabilitation goal, that is to enhance durable motor training effects in an aging population often exhibiting motor function decline.

## Author Contributions

GD, M-EB and LB: study conception and design. GD, M-EB, CC-P and MD: acquisition of data. GD and LB: analysis and interpretation of data, drafting of the manuscript. JD, DS-A: critical revision of the manuscript.

## Conflict of Interest Statement

The authors declare that the research was conducted in the absence of any commercial or financial relationships that could be construed as a potential conflict of interest.
